# Degradation of Decabromodiphenyl Ether Dispersed in Poly (Acrylo-Butadiene-Styrene) Using a Rotatory Laboratory Pilot Under UV-Visible Irradiation

**DOI:** 10.3390/molecules29215037

**Published:** 2024-10-25

**Authors:** Rachida Khadidja Benmammar, Zohra Bouberka, Christian Malas, Yvain Carpentier, Kawssar Mujtaba Haider, Venkateswara Rao Mundlapati, Michael Ziskind, Cristian Focsa, Skander Khelifi, Franck Poutch, Fouad Laoutid, Philippe Supiot, Corinne Foissac, Ulrich Maschke

**Affiliations:** 1 Unité Matériaux et Transformations (UMET), UMR 8207, CNRS, INRAE, Université de Lille, 59000 Lille, France; 2 Laboratoire Physico-Chimique des Matériaux, Catalyse et Environnement (LPMCE), Université des Sciences et de la Technologie d’Oran «Mohamed Boudiaf» (USTO-MB), Oran 31000, Algeria; 3 Institut Chevreul, CNRS, INRAE, Université de Lille, 59850 Villeneuve d’Ascq, France; 4 Physique des Lasers Atomes et Molécules (PhLAM), UMR 8523, CNRS, Université de Lille, 59000 Lille, France; 5 Department of Chemistry, School of Applied Science, Kalinga Institute of Industrial Technology (KIIT), Deemed to be University, Bhubaneswar 751024, India; 6 CREPIM, Rue Christophe Colomb, Parc de la Porte Nord, 62700 Bruay-la-Buissière, France; 7 Materia Nova Innovation Center, Avenue Copernic 3, 7000 Mons, Belgium

**Keywords:** e-waste, decabromodiphenylether, recycling, acrylonitrile-butadiene-styrene, UV-visible irradiation

## Abstract

The growing volume of plastics derived from electronic waste (e-waste) underscores the imperative for environmentally sustainable strategies for the management of this waste. In light of the paramount importance of this issue, a pilot demonstrator for the decontamination of polymers containing Brominated Flame Retardants (BFRs) has been developed. The objective is to investigate the potential for decontaminating BFR-containing polymers from e-waste via UV-visible irradiation using a rotatory laboratory pilot operating under primary vacuum conditions. This report focuses on binary model blends composed of 90 weight% (wt%) poly(Acrylo-Butadiene-Styrene) (ABS) pellets and 10 wt% Deca-Bromo-Diphenyl Ether (DBDE), which is one of the most toxic BFRs. The efficiency of the irradiation process was evaluated as a function of pellet diameter and irradiation time using Fourier Transform InfraRed spectroscopy (FTIR) and High-Resolution Laser Desorption/Ionization Mass Spectroscopy (HR-LDI-MS). As a consequence, ABS + DBDE achieved a decontamination efficiency of 97% when irradiated with pellets of less than 1 mm in diameter for a period of 4 h. Additionally, the thermal behavior of the irradiated samples was investigated through thermogravimetric analysis and differential scanning calorimetry. It was thus established that the application of UV-visible irradiation had no significant impact on the overall thermal properties of ABS.

## 1. Introduction

Polymeric materials have become an integral component of modern life, with a diverse range of applications in both the domestic and industrial sectors [1]. It is estimated that the global production of plastics will reach approximately 1.1 billion tons by 2050 [2].

The most recent statistics published by the United Nations indicate that 62 Mt of electronic waste is produced globally on an annual basis. Nevertheless, only 25.3% of this waste is documented to be collected and properly recycled. The remainder is incinerated or landfilled. However, the high energy consumption and cost of the landfill process mean that this waste is frequently released into water bodies [3,4].

In the context of the global transition to a safe and circular economy (CE), a number of research findings have influenced the European Union’s (EU) innovation policy, which oversees the implementation of CE principles in the waste electrical and electronic equipment (WEEE) industry [2]. With an annual growth rate of 3–4%, WEEE represents the fastest growing single waste stream in the world. This exponential growth can be attributed to the advent of new and innovative electronic products and their pervasive integration into contemporary lifestyles [5]. In 2006, the European Commission published a roadmap for the Registration, Evaluation, Authorization and Restriction of Chemicals (REACH EC1 907/2006), which serves as a tool for classifying chemically hazardous substances, in particular flame retardants (FRs) contained in WEEE [6]. Moreover, additional brominated FR (BFR) substances are continually added to the REACH/ROHS list [7,8]. Nevertheless, a multitude of manufactured goods (including household furniture, vehicles, textiles, and electronics) employ BFRs to enhance the intrinsic properties of polymers, thereby reducing their flammability in the event of combustion [9].

For over a decade, the recycling of plastic waste containing BFRs without decontamination has been banned by the EU due to the implementation of strict regulations. However, the incineration process, which is employed as an alternative method for the elimination of BFRs, is more detrimental to the ecosystem due to the generation of hazardous by-products. The residual ash from plastic waste incineration contains toxic elements, including sulfur, chromium, zinc, arsenic, selenium, mercury, and lead [10]. Consequently, the practice of plastic waste incineration gives rise to considerable concerns in relation to the environment and human health. Furthermore, it represents a considerable economic and energy loss over time.

The global annual consumption of BFRs has been estimated at approximately 680,000 tons in 2015, with projections indicating an anticipated increase to over two million tons by 2025 [11]. The degradation and leaching of BFRs have the potential to result in the contamination of various environmental compartments, including air, water, and soil [12]. The primary routes of exposure to BFRs for humans are inhalation and ingestion, as evidenced by a review of existing research [13]. The half-lives of BFRs in the human body have been estimated to range from 1 to 12 years [14,15,16,17,18,19].

In order to enhance the efficiency of BFRs, relatively high concentrations are typically employed in polymers, with 10 weight% (wt%) being the most prevalent for one single BFR molecule [20]. In many instances, the incorporation of a synergist, such as antimony trioxide (ATO), is essential for the optimal performance of these compounds. This compound is typically incorporated into plastic formulations at concentrations of 3–6 wt% (based on the weight of the plastic) in conjunction with halogenated FRs [21,22]. The use of BFRs in conjunction with ATO as a synergist is of paramount importance for certain applications, particularly in the case of Poly(acrylonitrile-butadiene-styrene) (ABS), which is one of the most preferred polymers for the fabrication of EEE enclosures from both a technical and economic perspective. Elevated concentrations of BFRs, including DecaBromoDiphenylEther (DBDE), were identified in human serum with a concentration of 36 ng/g lipid [23]. One of the most prevalent BFRs, DBDE, has been classified as a POP (Persistent Organic Pollutant) and is subject to global restrictions due to its chemical, physical, and biological resistance to degradation [24].

Nevertheless, recent studies have demonstrated the feasibility of purifying polymers from BFRs. A variety of solvent-based methods have been evaluated for the debromination of plastics derived from WEEE [25]. These include single-stage and two-stage pyrolysis, with the objective of optimizing the pyrolysis products obtained. In the initial stage, a concentration of bromine exceeding 20.1 wt% was observed, while a quantity of less than 2.0 wt% was identified in the subsequent phase. This resulted in the production of a high-quality liquid fraction with a low bromine content [26].

An investigation utilizing a supercritical solvent demonstrated a decontamination efficiency of approximately 43% for ABS containing TetraBromoBisPhenol A (TTBPA) [27]. The solvent, carbon dioxide (sc-CO_2_), exhibits characteristics of both liquids and gases, which confer high diffusivity and solubility.

A decontamination technology has been developed on an industrial scale (CreaSolv), which allows for the selective dissolution of e-waste and the thorough purification of the resulting polymer solution using mechanical and extractive approaches [28]. In addition to the criterion of solubility, the solvent must also be environmentally friendly, pose minimal risk to health and safety, require minimal energy for production, transportation, storage, use, and disposal, and be derived from renewable raw materials, which introduces further complications [29,30,31].

European initiatives have set forth a goal of increasing the proportion of recycled plastics in newly produced virgin plastics to reach 10 million tons by 2025. Nevertheless, several obstacles remain to be overcome, including the elevated cost of recycled polymers, their inferior quality in comparison to that of virgin polymers, and the complexity of recycling polymers that have been contaminated or rendered hazardous. The process presented in this report is particularly concerned with the decontamination of plastic waste, thereby increasing the proportion of reusable plastics and facilitating the transition to a circular economy.

Prior research has demonstrated the feasibility of decontaminating ABS and poly(carbonate) containing DBDE without the use of solvents through the application of UV-visible or electron beam (EB) irradiation [32,33,34]. These studies were conducted on thin polymeric films with a thickness ranging from 30 to 50 μm. The assessment of polymer characteristics following irradiation indicated the occurrence of crosslinking phenomena, accompanied by an acceleration in the presence of DBDE. Nevertheless, the utilization of a static low-pressure reactor for UV-visible irradiation and the presence of a nitrogen atmosphere throughout the EB process serve to prevent extensive photooxidation. The results obtained from thin films indicate that the irradiated polymer/BFR mixtures can be incorporated into pristine polymers without compromising their mechanical (cf. Figure 8 and Table 1 in [33]) and thermal (cf. Figure 7 in [33] and Figure 9 in [34]) properties. These findings substantiate the potential for valorizing polymer/BFR waste following exposure to irradiation processes. The irradiation-based processes can be considered economically viable, environmentally friendly (no waste, solvent-free), and capable of ensuring the necessary technical performance for recycled materials.

The objective of this report is to present the development of a rotatory laboratory pilot system designed for the decontamination of real plastic waste, which is typically available in the form of pellets within industrial settings. The decontamination process is conducted under UV-visible irradiation, with a wavelength range of 300 to 700 nm, and with the application of a primary vacuum. The plastic pellets, which have been selected to have diameters within a range from 0.3 to 3 mm, are placed in a rotating fused silica glass tube, which allows the penetration of UV-visible light.

In this investigation, a model polymer/BFR mixture (90 wt% ABS [35] + 10 wt% DBDE) was employed to examine the degradation efficiency of DBDE through the use of Fourier Transform InfraRed analysis (FTIR) and High-Resolution Laser Desorption/Ionization Mass Spectroscopy (HR-LDI-MS). This study focuses in particular on the effect of pellet dimension and irradiation time. Thermal Gravimetry Analysis (TGA) and Differential Scanning Calorimetry (DSC) were employed to monitor the thermal properties of the ABS + DBDE blend as a function of UV-visible exposure time.

## 2. Results and Discussions

### 2.1. Pellet Size Dependence on the Photodegradation Efficiency of DBDE

**Figure 1:**
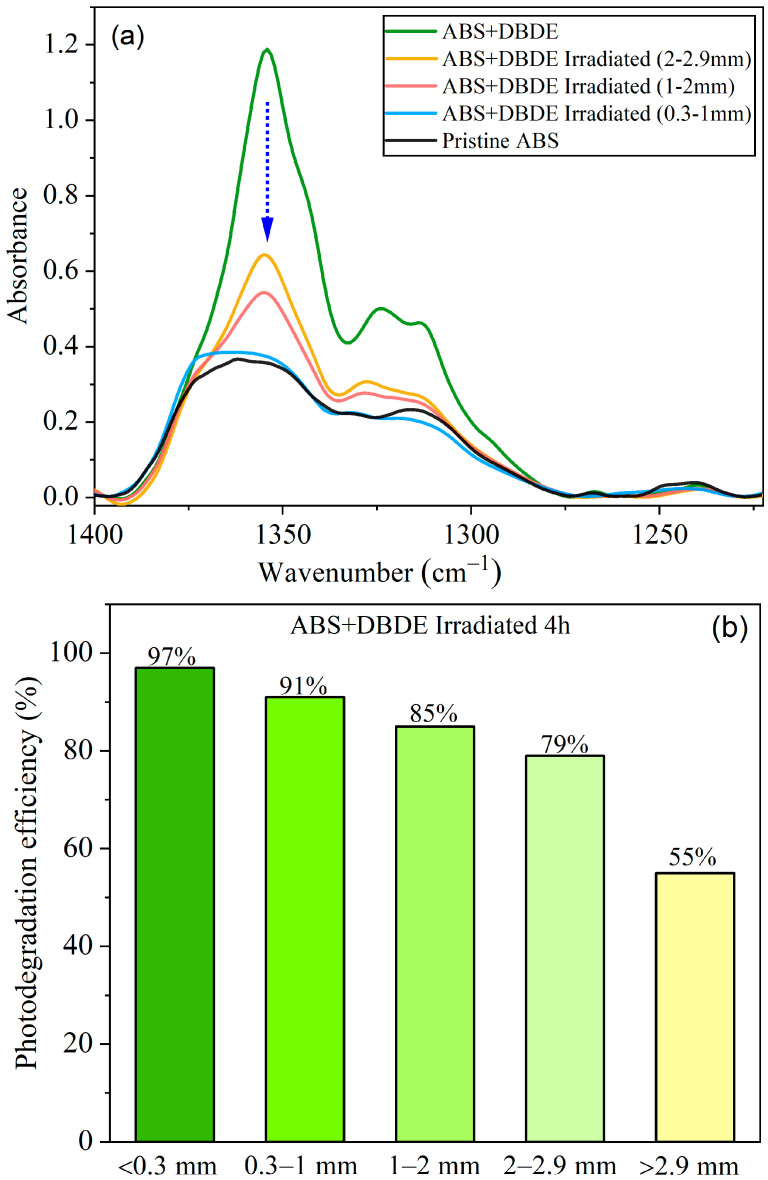
FTIR analysis of ABS + DBDE after 4 h of irradiation, as a function of pellets diameters: (**a**) Evolution of the aromatic C-O-C vibration band of DBDE, (**b**) photodegradation efficiency (in %) calculated from the integrated peak area between 1400 and 1250 cm^−1^.

The ABS + DBDE pellets, exhibiting a diameter range from 0.3 mm to 3 mm, were separated using sieves. To determine the photodegradation efficiency of DBDE, the separated pellets (0.3–1 mm, 1–2 mm, 2–2.9 mm) were subjected to UV-visible irradiation applying the experimental conditions described in Section 3. The subsequent section presents the results of a four-hour exposure conducted in dynamic mode. The results of the FTIR analysis indicated a significant reduction in the intensity of the DBDE vibration bands, which was found to be dependent on the diameter of the pellets (see Figure 1).

Specifically, DBDE displays a prominent and broad band spanning the range between and 1400 and 1250 cm^−1^, which corresponds to asymmetric C-O-C stretching vibrations in the aromatic rings [36]. This band of the ether function, which is not affected by other bands in the DBDE spectrum, can thus be selected as a means of monitoring the photodegradation of DBDE.

A more detailed analysis of the evolution of the intensity of the characteristic absorption bands corresponding to the latter vibration and those of the aromatic C-Br bands of DBDE revealed an inverse relationship between the photodegradation efficiency and the pellet diameter. Figure 1a illustrates this correlation for the C-O-C band. This suggests that larger pellets result in a lower degree of photodegradation than smaller pellets. This relationship is illustrated in Figure 1b, in which the DBDE photodegradation efficiency (expressed as a percentage) was calculated using the following formula: (A_0_ *−* A_T_)/A_0_ × 100, based on the integrated peak area between 1400 and 1250 cm^−1^ (C-O-C vibration) of DBDE. In this context, A_0_ and A_T_ correspond to the non-irradiated and irradiated films, respectively. Additionally, A_T_ is provided as a function of pellet thickness. The aforementioned expression has been adjusted by subtracting the peak area of the pristine ABS. In the case of pellets with a diameter of less than 1 mm, the C-O-C band is almost completely abated, and the photodegradation efficiency reaches 97%. The reduction in pellet size allows for greater penetration of UV-visible radiation, which results in the near-complete decontamination of ABS.

### 2.2. Monitoring the Degradation of DBDE

#### 2.2.1. Infrared Spectroscopy Analysis

**Figure 2:**
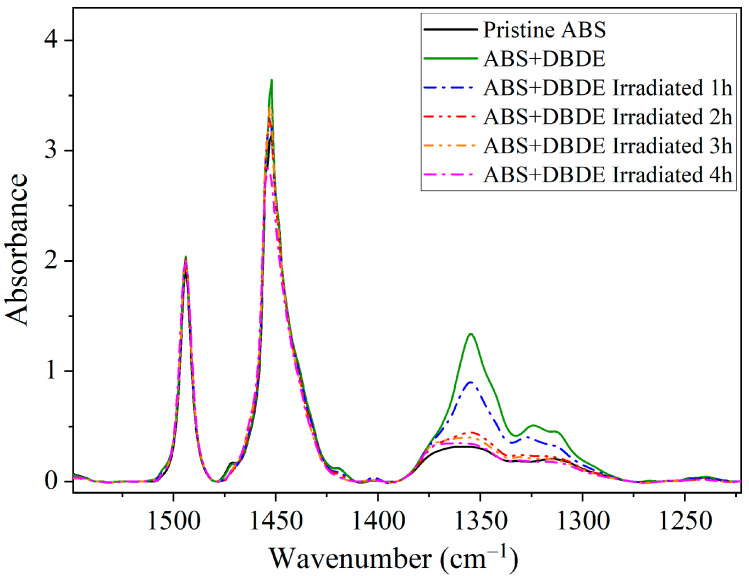
FTIR spectra of ABS + DBDE as a function of exposure time to irradiation in the range 1600–1200 cm^−1^: Evolution of the C-O-C ether band of DBDE between 1400 and 1250 cm^−1^.

The findings presented in this section pertain to small ABS + DBDE pellets (less than 1 mm in diameter) that were selected due to their high photodegradation efficiency by UV-visible irradiation. In this infrared spectroscopy analysis, several distinct absorption bands were employed to monitor the photodegradation of DBDE, corresponding to the C-O-C and C-Br vibrations. Figure 2 shows the FTIR spectra of the original ABS material and of ABS samples containing DBDE, both irradiated and non-irradiated, within the range of 1600–1200 cm^−1^. This figure illustrates the reduction in the intensity of the C-O-C band of DBDE (range 1400–1250 cm^−1^) as the duration of radiation exposure increases. Following a two-hour period of light exposure, the C-O-C band exhibited a pronounced decline, and after four hours, it had almost completely disappeared, indicating a significant reduction in the amount of DBDE present.

The absorption band observed at 1453 cm^−1^ can be attributed to a CH_2_ planar deformation, while the band at 1494 cm^−1^ can be associated with the C=C stretching vibration in the aromatic rings of the styrene component of ABS, in accordance with reference [37]. 

Figure 2 illustrates a decline in the intensity of the absorption band at 1453 cm^−1^ as the exposure time to UV-visible irradiation increases. This phenomenon is associated with a reduction in the number of CH_2_ groups, which are predominantly located in the polymer backbone. Conversely, the absorption band at 1494 cm^−1^ was shown to remain constant during irradiation, indicating that the aromatic rings of the styrene part of ABS have not been affected.

**Figure 3:**
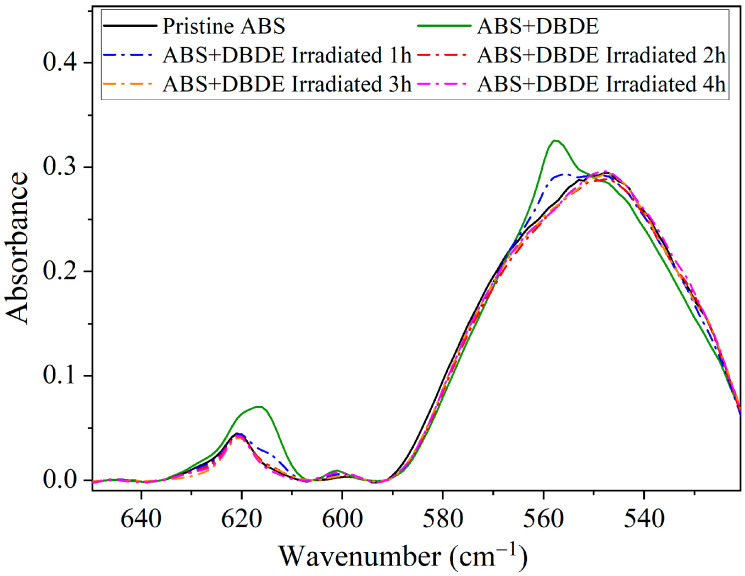
FTIR spectra of ABS + DBDE in the range 650–520 cm^−1^ as a function of irradiation time: evolution of the aromatic C-Br bands of DBDE between 550 and 565 cm^−1^ and 605 and 635 cm^−1^.

Figure 3 depicts the FTIR spectra of ABS + DBDE as a function of irradiation time within the wavenumber range 650–505 cm^−1^. The intensity of the absorption bands corresponding to the aromatic C-Br stretching vibrations of DBDE, observed at 617 [38] and 557 cm^−1^ [39,40], exhibited a pronounced decline as a function of irradiation time. Indeed, after a period of two hours of UV-visible light exposure, the intensities of these two absorption bands were found to be similar to those of the pristine ABS, indicating that bromine was almost completely eliminated from carbon-containing species.

**Figure 4:**
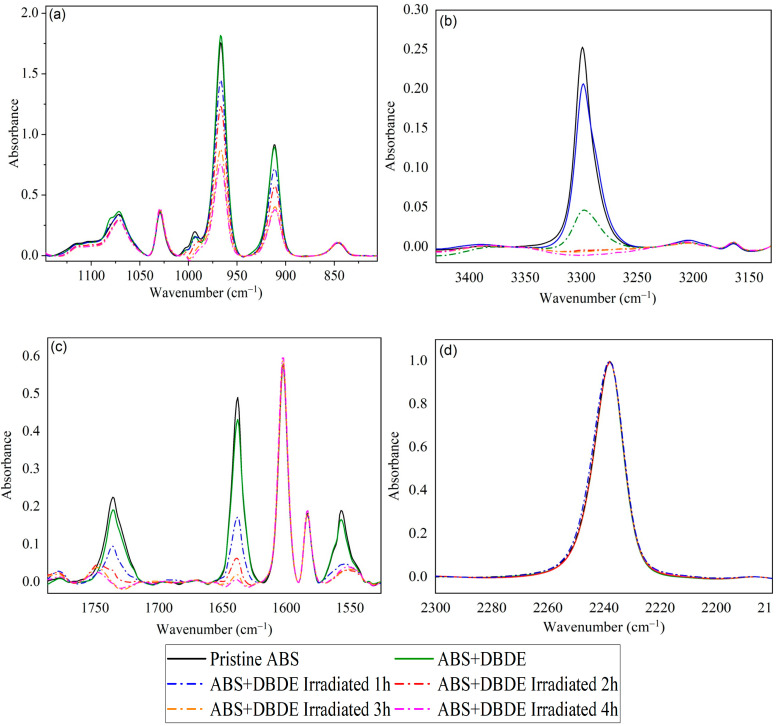
FTIR spectra of ABS + DBDE as function of irradiation time: (**a**) C-C bands between 1150 and 860 cm^−1^, (**b**) N-H bands of polymer additives between 3350 and 3240 cm^−1^, (**c**) C=N bands of polymer additives between 1775 and 1525 cm^−1^, (**d**) C≡N band of ABS at 2237 cm^−1^.

Figure 4a provides further evidence that the butadiene component of the ABS triblock copolymer demonstrates heightened vulnerability with increasing duration of UV-visible irradiation. In particular, this figure illustrates the presence of two principal absorption bands, situated between 890 and 940 cm^−1^ and between 940 and 990 cm^−1^, respectively. The initial band is not present in the DBDE spectrum, while an absorption band at 960 cm^−1^ is attributed to a C-Br vibration [36]. In contrast, the ABS spectrum exhibits two bands at 911 and 966 cm^−1^, which correspond to the stretching vibrations of vinyl CH_2_ and trans C-H, respectively, in the butadiene component [41]. These latter two absorption bands demonstrate a reduction in intensity with increasing irradiation periods, thereby corroborating the impact of irradiation, particularly on the butadiene component of ABS, which may result in a cross-linking effect of the polymer. A solubility study was subsequently conducted in an organic solvent (tetrahydrofuran, THF) to ascertain the percentage of soluble and insoluble components in the irradiated samples. It should be noted that the pristine ABS sample exhibited linear chains that were fully soluble in THF. The insoluble portions of the irradiated samples were found to comprise, on average, 3 wt%, which was attributed to the cross-linked polymer. Therefore, FTIR analysis could not confirm the presence of cross-linking species.

The ABS utilized in this study contains a variety of polymer additives, including chemical blowing agents, lubricants, and antistatic agents that contain nitrogen groups. In particular, antistatic agents, such as ammonium salts or ethoxylated amines, contribute to the formation of nitrogen-bonded bands. Furthermore, azoamides are utilized as blowing agents for ABS, including azodicarbonamide, which exhibits N-H absorption bands at 3300, 1735, 1640, and 1555 cm^−1^, analogous to Figure 4b,c [42].

Spectral analysis of slip agents with N-H and C=N bands around 3300, 1637, and 1556 cm^−1^ (e.g., long-chain fatty acid amides) reveals contributions to the IR spectrum [43]. It can be proposed that these polymer additives tend to dissipate following exposure to radiation. Consequently, the recycling of polymers must adhere to the same process as that of a new product, namely the re-addition of new additives. Figure 4d demonstrates that the C≡N band of the acrylonitrile group, situated at 2237 cm^−1^, remained unaltered by the irradiation, indicating a notable stability of the acrylonitrile component of the triblock copolymer.

**Figure 5:**
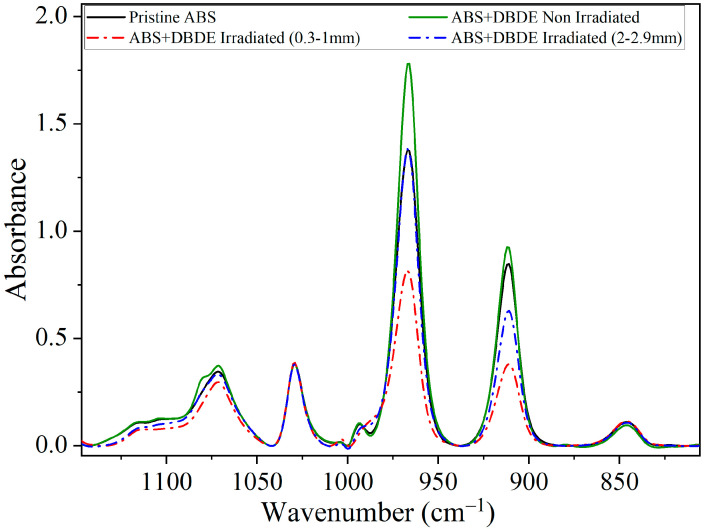
FTIR spectra of pristine ABS, ABS + DBDE and irradiated ABS + DBDE (two pellet thicknesses, 4 h of irradiation), obtained in the range between 805 and 1190 cm^−1^.

Furthermore, the granulometric study indicated that the butadiene component of ABS, which is the portion of this polymer most susceptible to irradiation effects, as previously demonstrated, exhibited a significantly reduced impact from irradiation for 2 mm diameter pellets compared to those with diameters smaller than 1 mm. This is demonstrated in Figure 5, which illustrates that the spectra of irradiated ABS + DBDE pellets with a diameter of 2 mm are nearly superposed with that of pristine ABS in the range of wavenumbers from 1190 to 805 cm^−1^, with the exception of a slight impact on the intensity of the CH_2_ stretching vibration at 911 cm^−1^ (butadiene component). In contrast, as illustrated in Figure 1, the DBDE molecule exhibited incomplete abatement, with abatement levels reaching 79% for pellets with a diameter of 2 mm.

#### 2.2.2. High-Resolution Laser Desorption/Ionization Mass Spectroscopy Analysis

**Figure 6:**
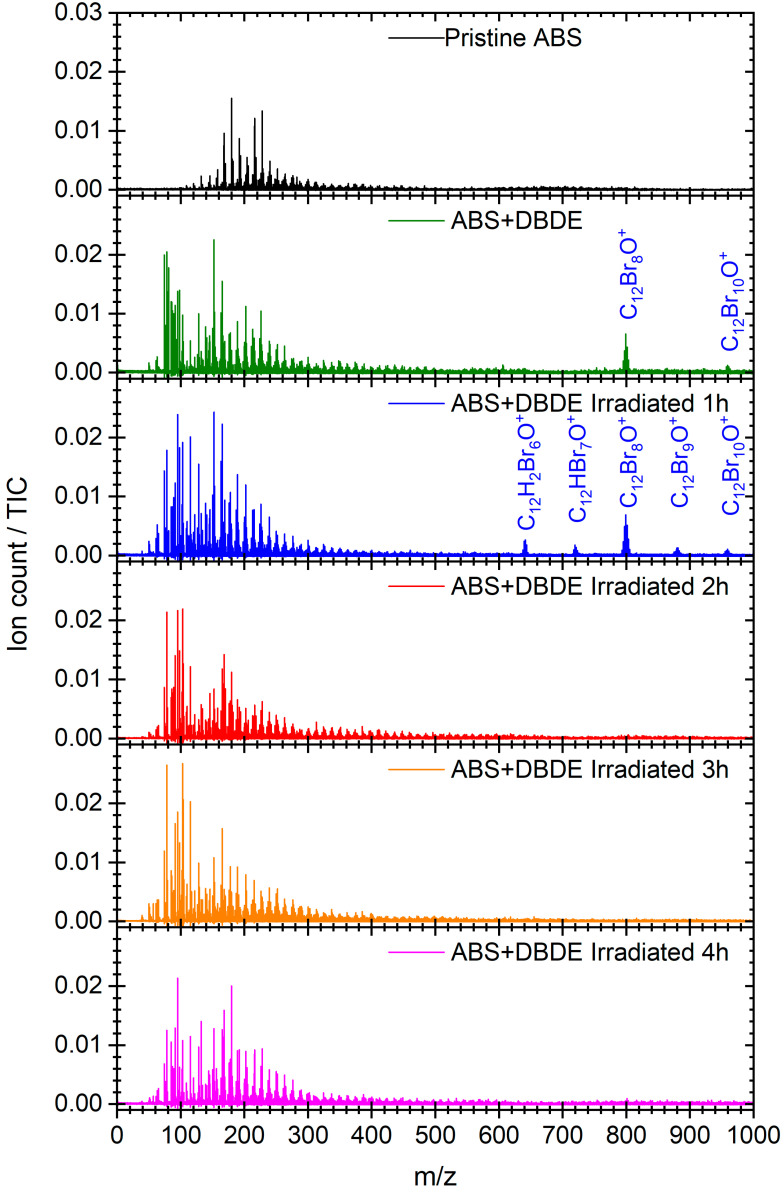
The mass spectra of “Pristine ABS”, “ABS + DBDE”, and “ABS + DBDE Irradiated x h” were subjected to four distinct UV-Vis irradiation durations (x = 0–4 h). The main species related to diphenylether compounds are identified and indicated. The spectra have been normalized to the total ion count (TIC).

**Figure 7:**
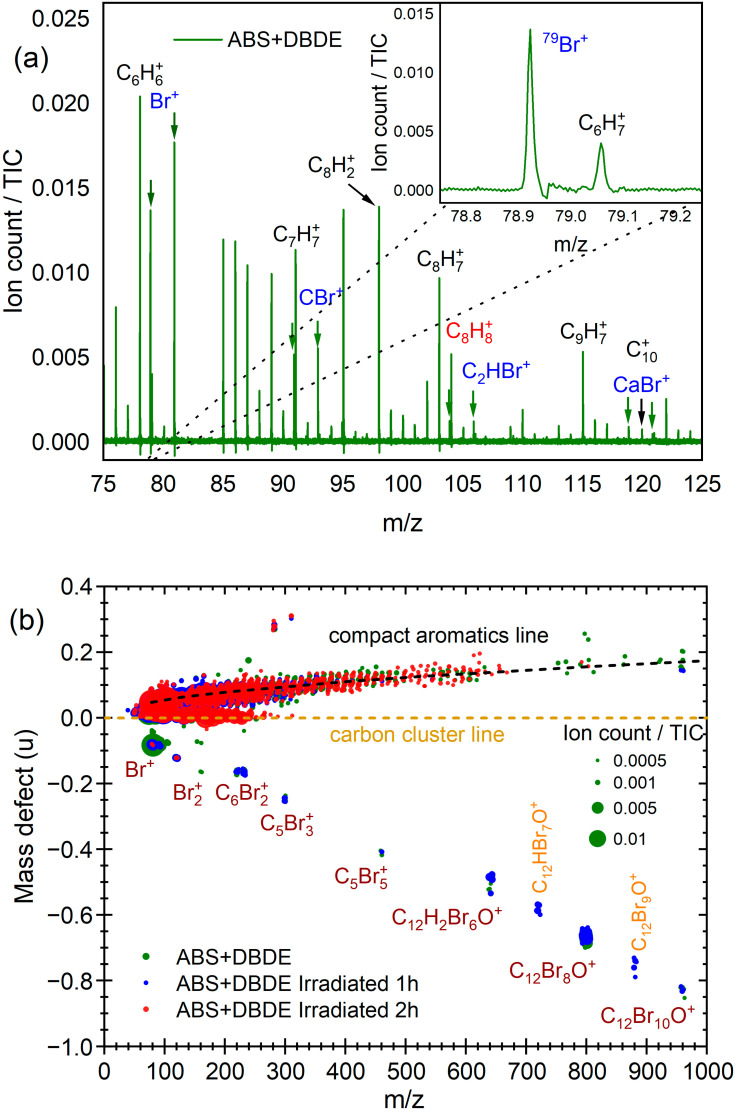
(**a**) Mass spectrum of ABS + DBDE within the *m*/*z* = 75–125 region, which reveal the presence of brominated fragments; (**b**) Mass defect plots of ABS + DBDE (green) and ABS + DBDE Irradiated 1 h (**blue**) and 2 h (**red**). The area of the bubbles is proportional to the mass intensity signal relative to the total ion count (TIC).

The mass spectra for the pristine ABS, the untreated ABS + DBDE, and the ABS + DBDE subjected to four distinct durations of UV-Vis irradiation are presented in Figure 6. In the low-mass region (*m*/*z* < 400), the spectra are mainly characterized by the presence of hydrocarbon fragments derived from the plastic polymer. The spectra of the samples that originally contained DBDE exhibited smaller hydrocarbon and brominated fragments (*m*/*z* < 110), including Br^+^, CBr^+^, C_2_HBr^+^, and the styrene-derived species C_8_H_7_^+^ (*m*/*z* 103.055). For further details, please refer to Figure 7a. At higher mass, the ABS + DBDE sample exhibits isotopic distributions of DBDE species around *m*/*z* = 959.17 and compounds containing high bromine atoms around *m*/*z* = 799.33. The overwhelming majority of the mass peak series around *m*/*z* = 799.33 can be attributed to the fragmentation of DBDE under the influence of the ablation laser at 532 nm rather than octabromodiphenyl ether (OBDE), which is anticipated to be present in conjunction with DBDE. In the ABS + DBDE spectrum obtained after one hour of UV-Vis irradiation, the presence of additional DBDE and OBDE fragments is discernible. These include diphenyl ethers containing six, seven, and nine bromine atoms. This observation of additional fragments is linked to a debromination process of the parent species DBDE and OBDE during the first two hours of UV-Vis irradiation. For the three irradiation times of ≥2 h, neither DBDE nor its aforementioned fragments at *m*/*z* > 400 are observable, indicating the efficiency of the laboratory pilot in dissociating brominated species of at least six bromine atoms.

In order to ascertain the presence of small brominated fragments in the ABS + DBDE sample (green bubbles), or to confirm their absence following UV-Vis irradiation, particularly in the region comprising predominantly hydrocarbon fragments (see Figure 7a), a mass defect plot was created, presented in Figure 7b. In this type of plot, achieved thanks to the excellent mass resolution of the instrument (m/Δm > 10,000), each mass peak in the spectrum is represented by a bubble whose area is proportional to the intensity of the peak and whose position is marked on the horizontal axis by the mass-to-charge ratio (*m*/*z*) and on the vertical axis by the mass defect (here defined as the difference between the measured mass and the nucleon number of the assigned species). Chemical species that vary ^12^C atoms, such as C_n_ carbon clusters, are aligned horizontally. In contrast, species that differ only in their hydrogen atom content (*m*/*z*(^1^H) = 1.007825) follow a slope of 0.07764. The brominated species (*m*/*z*(^79^Br) = 78.918 and *m*/*z*(^81^Br) = 80.916) are distinguished by significant negative mass defect values.

The laser fluence was selected to yield the formation of small brominated species, as observed in the untreated ABS + DBDE sample. In light of these conditions, it is reasonable to anticipate the observation of all brominated species fragments produced as a consequence of UV-Vis irradiation.

In the case of ABS + DBDE, the mass defect representation allows for the straightforward identification of bromine-containing species (*m*/*z* < 400) inherent to the laser analysis method and chosen fluence. The following species were identified: Br^+^, CBr^+^, C_2_HBr^+^, CaBr^+^, Br_2_^+^, C_6_Br_2_^+^, and C_5_Br_3_^+^. The sample that underwent one hour of UV-Vis irradiation (blue bubbles) exhibited the same small fragments, along with C_12_HBr_7_O^+^ and C_12_Br_9_O^+^. Following a two-hour irradiation period, the aforementioned phenomena are no longer observable, with the exception of fragments containing less than two bromine atoms, which exhibit a markedly diminished intensity. This finding corroborates previous infrared spectroscopic analyses of the efficiency of the laboratory pilot in near-complete decontamination of ABS + DBDE after two hours of irradiation.

### 2.3. Thermal Properties

#### 2.3.1. Thermogravimetry

**Figure 8:**
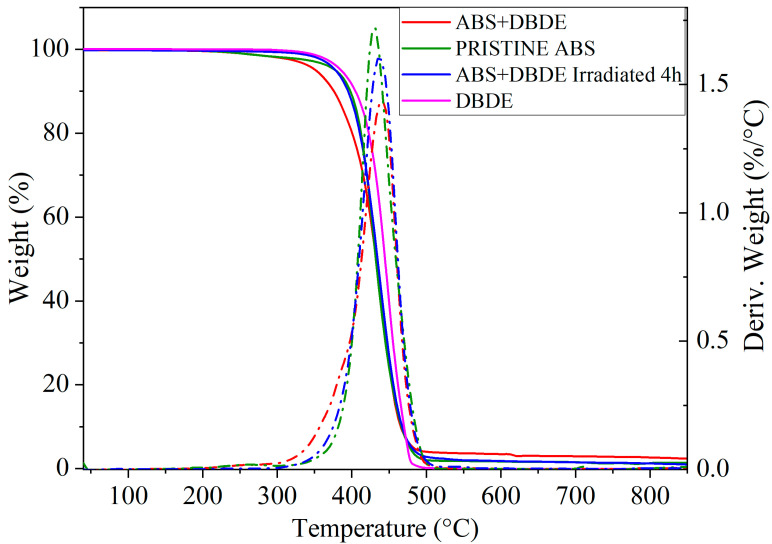
Weight losses (continuous lines) and their derivatives (dashed lines) obtained by TGA for pristine ABS, ABS + DBDE, and irradiated ABS + DBDE.

The following sections of this report will focus on the degradation behavior of irradiated (4 h) ABS + DBDE samples with a pellet diameter below 1 mm. These samples are more susceptible to significant photodegradation of DBDE due to their small diameter, which is directly related to the penetration depth of the wavelengths. At the same wavelength, the smaller the pellet diameter, the greater the penetration depth of the light. The resulting TGA and DTA curves are presented in Figure 8. The decomposition profiles of these samples demonstrate that thermal degradation occurs in a single step for all cases at a heating rate of 10 °C/min, indicating that there is a single predominant degradation reaction. Some studies have observed a single degradation step, while others have observed two degradation steps, which can be attributed to the crosslinking of the polymer [34,44].

Figure 8 illustrates that the thermal degradation of pristine ABS commences at approximately 406 °C, which corresponds to T_onset_. The maximum rate of weight loss is observed at 428 °C. At 900 °C, approximately 0.4 wt% of the initial weight remains, which is likely composed of inorganic charges. The thermogram of the pristine DBDE exhibits a T_onset_ at 412.9 °C, which is higher than that observed for the pristine ABS.

However, the addition of DBDE to ABS results in a reduction in thermal stability compared to that of the pristine ABS, with T_onset_ = 396.4 °C. This may be attributed to a plasticizing effect, which consists of an increase of free volume between the polymeric molecules. Subsequently, a degradation step was observed at a temperature slightly higher than that found for the virgin ABS, with a maximum mass loss occurring at 437.5 °C. A residual weight of 2% was observed at 900 °C. The irradiated ABS + DBDE displays a T_onset_ that is similar to that observed in the pristine ABS. The maximum mass loss occurs at 437.6 °C, representing an increase of 9 °C compared to the pristine ABS. This is likely due to the presence of cross-linked species. The findings of this study indicate that the degradation profile of irradiated ABS + DBDE is analogous to that of pristine ABS. The primary findings are summarized in Table 1.

#### 2.3.2. Differential Scanning Calorimetry Analysis

**Figure 9:**
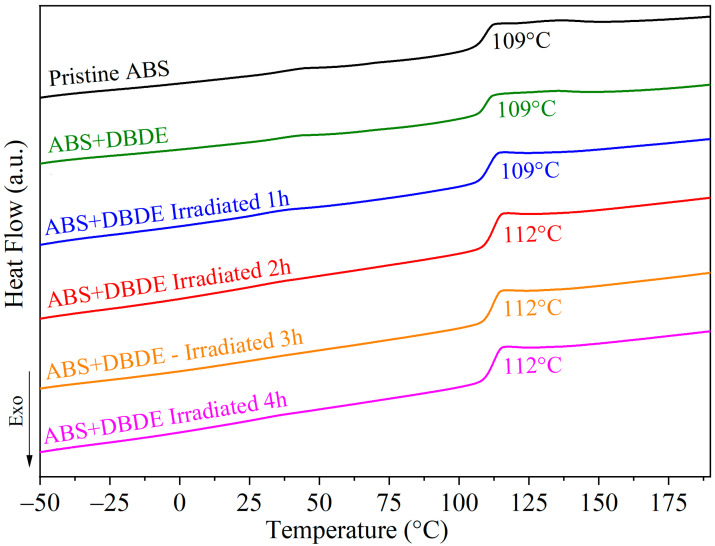
DSC thermograms of pristine ABS, ABS + DBDE and irradiated ABS + DBDE at different exposure periods, corresponding to the second heating cycle.

The thermal properties of ABS + DBDE samples (pellet diameter < 1 mm) were investigated through the utilization of DSC measurements, both prior to and following the decontamination process. Figure 9 depicts the thermograms obtained for pristine ABS, ABS + DBDE, and irradiated ABS + DBDE at varying exposure times to UV-visible light. Given that ABS is an amorphous polymer, a single glass transition (T_g_) can be anticipated from DSC measurements. Indeed, a single T_g_ was observed for all samples analyzed, irrespective of the irradiation time. The incorporation of DBDE into the virgin ABS matrix does not result in a change to the T_g_ of the polymer, which remains at 109 °C.

As illustrated in Figure 9, the irradiated ABS + DBDE samples exhibit a T_g_ of 112 °C, which represents a 3 °C increase compared to the pristine ABS and ABS + DBDE samples. This increase in T_g_ is attributed to a weak cross-linking effect, which results in a marginal enhancement of the thermal stability of the pristine ABS [45]. This cross-linking effect can be linked to photodegradation effects on DBDE, additives, and particularly the butadiene part of ABS. The formation of stable free radicals by the dissociation of carbon bands does not result in migration along the polymer chains. Conversely, the migration of polymer radicals facilitates their evolution toward cross-linking reactions.

Moreover, a minor endothermic effect was discerned between 40 and 50 °C for the virgin ABS and unirradiated ABS + DBDE, which can be attributed to the melting of the additives [46]. This phenomenon dissipates after four hours of irradiation. These findings are in accordance with the results of the FTIR analysis (cf. Figure 4).

## 3. Experimental Section

### 3.1. Materials

The DBDE was procured from Green Chemical S.p.A., an Italian enterprise headquartered in Desio. The chemical purity of the DBDE is 98%, its molecular weight corresponds to 959 g/mol, and its melting point is above 300 °C. The operational temperature of the DBDE is maintained below its degradation temperature, as its use in dynamic mode precipitates the acceleration of the degradation temperature to a lower temperature. The ABS was obtained from AMP Polymix (Horbourg-Wihr, France) through Chi Mei Corporation (Tainan, Taiwan). The material exhibits a melt flow index of 4 mL/10 min, a mass density of 1.03 g/cm³, and an average molecular weight of 210,000 g/mol. An ^1^H-NMR study on deuterated chloroform was conducted to ascertain the proportion of each component of the triblock present in the ABS structure. This was achieved by analyzing the corresponding peaks from designated protons, which yielded the following results: Acrylonitrile: 30 ± 3%, Butadiene: 11 ± 3%, Styrene: 59 ± 5%.

Mixtures of 90 wt% ABS and 10 wt% DBDE were prepared using a Micro 15HT micro-extrusion machine, manufactured by Xplore Instruments BV (Sittard, Netherlands). The machine was equipped with a twin-screw extruder, which operates in dynamic mode, thereby accelerating the onset of thermal degradation of the polymer. Consequently, it is necessary to work at a lower temperature than that of the hot press, which operates in static mode. In order to accommodate the combined thermal and mechanical constraints that might alter some properties of ABS and DBDE, a reduction in temperature to 185 °C is required for the extrusion process. The preparation of small pellets was conducted at a temperature of 185 °C, with a pressure of 5 bar and a screw speed of 150 tr/min. The average diameter of the pellets was found to be approximately within the range of 0.3 to 3 mm. A series of test sieves was employed for the purpose of separating the pellets according to their respective diameters. The following five ranges were obtained: below 0.3 mm, between 0.3 and 1 mm, between 1 and 2 mm, between 2 and 2.9 mm, and above 2.9 mm.

### 3.2. Laboratory Pilot

The pilot apparatus is a laboratory-type apparatus exhibiting an internal volume of 1 L. It is constructed from aluminum and Teflon components. The device operates on a rotary basis, with a direction reversal occurring at intervals of five revolutions. The apparatus rotates at a rate of four revolutions per minute. A quantity of 1 g of pellets is introduced for the purposes of a single experiment. The entire internal atmosphere of the device is maintained under vacuum using a primary pump, with a pressure of approximately 10^−2^ mbar. The apparatus is composed of two silica glass tubes. The larger tube, measuring 11 × 16 cm^2^, is utilized to maintain the pilot under vacuum, while the smaller tube, measuring 8 × 13 cm^2^, serves as a rotating tube inside the first one, into which the polymer pellets are introduced. The use of fused silica tubes allows for high penetration of UV-visible light. Samples were irradiated for periods of 1, 2, 3, and 4 h. The experiment was conducted in a single step, with the irradiation periods not accumulating.

**Figure 10:**
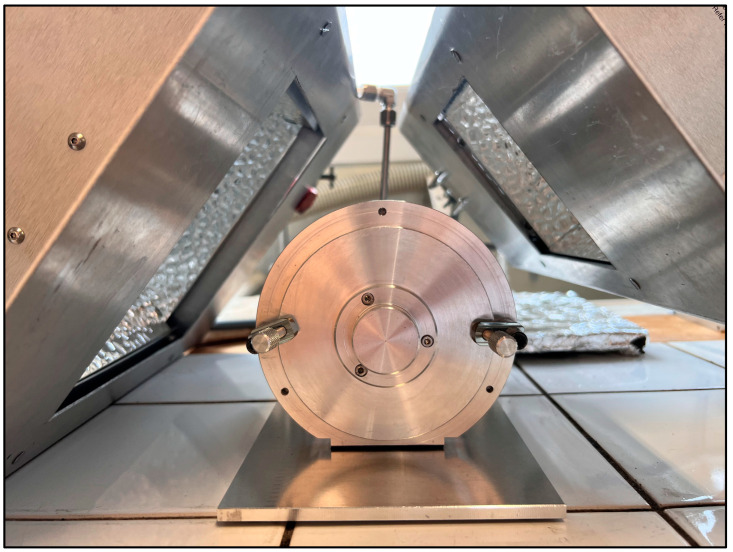
Front view of the vacuum-packed rotary pilot exposed parallel to the light sources.

Two industrial-type light sources (Dymax Europe GmbH, Wiesbaden, Germany) were positioned in parallel to the silica tubes at a distance of approximately 10 cm. One of the light sources displays more intense bands within the visible range (360–700 nm), which permits greater penetration of the radiation into the pellets. The other source exhibits a wavelength range situated between 300 and 600 nm, which is more powerful in terms of energy. The lamps were positioned in order to ensure an even distribution of radiation across the entire volume of the silica tubes. These Dymax light sources are designed for use in a wide range of production environments [47]. The temperature generated by this type of lamp can be regulated by maintaining a specific distance between the control apparatus and the lamps, allowing for temperature control. A photograph of the laboratory pilot together with the UV-visible light sources is provided in Figure 10 for the reader’s convenience.

### 3.3. Fourier Transform Infrared Spectroscopy

The degradation of DBDE was monitored using FTIR in order to analyze the structural changes that occurred in the polymer both before and after irradiation. The analysis was conducted using a PerkinElmer Frontier spectrometer (Perkin Elmer, Waltham, MA, USA). A total of 16 scans were accumulated, with a spectral resolution of 4 cm^−1^. The analysis was conducted within the spectral range of 4000 to 500 cm^−1^ at room temperature. To facilitate acquisition in transmission mode, the polymer pellets were converted into films of ABS and ABS + DBDE, with thicknesses ranging from 30 to 50 µm. A laboratory-based molding press was utilized to produce thin films. A laboratory press manufactured by Servitec Maschinenservice GmbH (Berlin, Germany) was utilized. The device was a Servitec Polystat 200T. The press was operated with 90 bars applied gradually over a four-minute period at a temperature of 230 °C. The collected spectra were baseline-corrected and normalized to a specified wavenumber of pristine ABS.

### 3.4. Thermogravimetric Analysis

Thermogravimetric analysis (TGA) was conducted on the samples using a TA Discovery instrument (Guyancourt, France). Samples with an average mass of 8 mg were prepared in platinum pans and processed in a nitrogen environment with a heating ramp of 10 °C/min and a gas flow of 50 mL/min. The polymer was subjected to processing at temperatures spanning a range of 25 to 900 °C. The temperature at which a deviation is first observed is referred to as the T_onset_, while the temperature at which the process ends is known as the T_endset_.

### 3.5. Differential Scanning Calorimetry

The glass transition temperatures of both the unirradiated and irradiated samples were determined using a DSC Q2000 instrument from TA Instruments (New Castle, DE, USA). The samples were prepared by placing 9 ± 3 mg of the polymers in an aluminum crucible. The heat flow data were normalized to the weight of the samples. The heating and cooling rates were maintained at 10 °C/min within the temperature range of −70 to +200 °C, under nitrogen flow and a gas flow of 50 mL/min. At the outset of the experiment, the sample was cooled, and three subsequent heating and cooling cycles were conducted to account for any thermal events associated with the sample preparation history. The thermograms subjected to analysis in this study were obtained from the second heating cycle. To ensure the reproducibility of the results, at least three experiments were conducted on identical samples with the same composition and independent preparation. The glass transition temperatures were determined by calculating the midpoint within the transition range of the thermograms, as previously described.

### 3.6. High-Resolution Laser Desorption/Ionization Mass Spectroscopy

Polymer pellets, selected in diameter size (0.3–0.9 mm) and processed into films, were subjected to analysis by high-resolution laser desorption/ionization mass spectrometry (HR-LDI-MS) in accordance with a comprehensive experimental setup previously detailed elsewhere [48]. The film, with a thickness of 30–50 µm, was positioned on a copper sample holder within a high vacuum chamber (residual pressure of 10^−7^ mbar). The sample was irradiated at normal incidence by nanosecond pulses from a frequency-doubled Nd:YAG laser (Quantel Brilliant EaZy, wavelength 532 nm, pulse duration 4 ns, repetition rate 10 Hz, energy per pulse 1.5 mJ). The laser beam was focused at a beam waist of 250 µm at the sample surface (corresponding fluence: ~3 mJ.cm^−1^), resulting in the ablation of charged compounds in a plume that expanded orthogonally to the film surface. Post-ionization was not employed in this study, as it did not result in an enhanced signal for brominated species. However, it remains a viable approach for the detection of aromatic compounds. Ion packets were extracted and directed into an ion trap, where they were thermalized by collision with helium atoms injected through a fast solenoid valve. Subsequently, the samples were subjected to analysis by a time-of-flight mass spectrometer (ToF-MS) equipped with a reflectron. The thermalization and shaping of the ion packet by ion optics yielded a mass resolution of m/Δm~10,000. The mass spectra of each film were obtained by accumulating 400 laser pulses across 10 mass windows, encompassing the full mass range (50–1000 *m*/*z*). During each scan, the sample was moved in its plane using Attocube’s piezoelectric nanopositioners to ensure that the next laser shot targeted a new point on the film. To ensure consistency of results, spectra from four distinct regions of the samples were acquired.

## 4. Conclusions

A laboratory rotary pilot apparatus was designed with the objective of decontaminating polymers containing toxic BFR without the use of solvents. The apparatus is equipped with a vacuum tube and irradiation sources that emit a wide range of UV and visible light. A model system was elaborated using ABS, a widely utilized industrial polymer, mixed with 10 wt% of DBDE, which is prevalent in e-waste deposits. The study was conducted on polymer pellets with diameters ranging from 0.3 to 3 mm. The findings indicated that the application of radiation was more efficacious in smaller samples, resulting in an enhanced decontamination of the model samples.

The disappearance of the brominated DBDE molecule was evaluated via FTIR spectroscopy, which demonstrated a high efficiency of approximately 97% for the C-Br band using ABS + DBDE pellets with a diameter of 0.3 to 1 mm and a continuous irradiation time of 4 h. These findings were corroborated by high-resolution mass spectrometry, which demonstrated that nearly complete removal of DBDE from the model mixture was achieved. The results of the thermal analysis, conducted using DSC and TGA, indicated that the ABS retained its thermal properties during the radiation exposure process.

## Figures and Tables

**Table 1:** Thermal properties obtained via TGA and DSC experiments (T_onset_: onset degradation temperature, T_endset_: endset degradation temperature, ΔT = T_endset_-T_onset_, T_max_= temperature where the rate of the degradation reached its maximum value, T_g_: glass transition).

Materials	TGA			DTG	DSC			
	T_onset_	T_endset_	ΔT	T_max_	T_onset_	T_endset_	ΔT	T_g_
	/°C	/°C	/°C	/°C	/°C	/°C	/°C	/°C
Pristine ABS	405.9	456.6	50.7	428	105.4	111.6	6.2	109.6
ABS + 10 wt. %DBDE	396.4	458.3	61.9	437.5	106.6	112.3	5.7	109.4
ABS + DBDE Irradiated 4 h	404.5	463.9	59.4	437.6	109	113.8	4.8	112

## Data Availability

The data presented in this study are available on request from the corresponding author.
